# The Impact of COVID-19 on Informal Caregivers’ Health Behaviors

**DOI:** 10.1093/geroni/igaa057.3505

**Published:** 2020-12-16

**Authors:** Mary Greaney, Zachary Kunicki, Megan Drohan, Steven Cohen

**Affiliations:** 1 University of Rhode Island, Kingston, Rhode Island, United States; 2 Brown University, Providence, Rhode Island, United States

## Abstract

The population of older adults aged 65+ in the US is projected to increase from 15% to 21% in the next 30 years. Aging in place provides cost-savings and familiarity to the older adult, but often requires informal caregivers. Informal caregivers, individuals who provide unpaid care of assistance to family members and friends may have been uniquely impacted by the COVID-19 pandemic and shelter-at-home orders. Research is needed to examine how the pandemic impacted caregivers’ caregiving responsibilities and health behaviors (e.g., physical activity, sedentary time, fruit and vegetable intake, snacking, etc.) as this information will be invaluable to determine if health promotion interventions are needed for informal caregivers. Self-reported data were gathered from informal caregivers providing care to someone aged 50+ (n=835) through Amazon’s Mechanical Turk. Respondents reported their current and pre-pandemic health behaviors and demographics. Chi-square tests were used to examine bivariate associations between pandemic time (pre vs. post) and each examined behavior. The analysis identified some positive health behavior changes due to the pandemic: caregivers felt since the pandemic they ate more fruits and vegetables (p < .001), walked more, exercised more (p < .001), increased amounts of sleep (p < .001), and higher sleep quality (p < .001). However, respondents also had more screen time (p < .001) and sedentary time (p < .001). Future planned analyses will focus on examining whether these changes were consistent across all sociodemographic subgroups of caregivers and whether they persist after the pandemic recedes.

